# Evolutionary and empirical perspectives on ‘demand’ breastfeeding: The baby in the driver’s seat or the back seat?

**DOI:** 10.1093/emph/eoae003

**Published:** 2024-01-20

**Authors:** David P Tracer

**Affiliations:** Department of Health and Behavioral Sciences, University of Colorado Denver, Denver, CO, USA; Department of Anthropology, University of Colorado Denver, Denver, CO, USA

**Keywords:** breastfeeding, demand feeding, parental investment, maternal–child health

## Abstract

**Background/Objectives:**

The concept of ‘demand’ breastfeeding is central in public health. A key feature of the concept is that the infant is the locus of control in the breastfeeding process; when the breast is demanded by the infant, it is given the opportunity to feed. This study questions this notion of the infant as the locus of control in demand breastfeeding for empirical and theoretical reasons. From an evolutionary perspective, infants are expected to seek maximal investment and, against this backdrop of maximal investment-seeking, parents decide how much investment to put into offspring.

**Methodology:**

Focal follows were conducted among 113 mother–infant dyads in Papua New Guinea. During these follows, response times and types of responses, including breastfeeding to offspring fussing and crying, were recorded.

**Results:**

Infants were breastfed an average of 3.6 times/hour for just over 2 min/feed. Fussing and crying were responded to quickly, with most response times under 1 min. When the mother responded, she breastfed the child approximately 52% of the time. The other 48% of the time, mothers responded to infants with other forms of pacification. Mothers were significantly less likely to respond to infants by breastfeeding if the child had been breastfed within the past 59–76 min.

**Conclusion/Implications:**

As predicted by evolutionary parental investment theory, infants make frequent demands on their parents for investment, but mothers are ultimately the locus of control in the investment process. The mother decides whether and how frequently to breastfeed her offspring against this backdrop of near-continuous investment demands.

## INTRODUCTION

The concept of ‘demand’ breastfeeding (sometimes ‘on-demand feeding’ or ‘response feeding’) is central in the public health and parenting literature. It is used to typify infant feeding patterns in many ‘traditional’ societies and to draw contrasts with the ‘scheduled’ infant feeding patterns that occur in most industrialized and urban contexts. Yet, despite its wide use in the literature, it remains a vague and ill-defined concept [1]. A key feature intrinsic to the concept is that when an infant fusses and/or cries, this acts as a cue to the mother that the breast is being demanded by the infant and, thus, it is offered the opportunity to feed. According to this notion, when ‘demand’ breastfeeding is practiced, the infant is the locus of control in the feeding process, governing both its frequency and duration. This article provides a brief review of the history of the term ‘demand’ breastfeeding, its definition and use in the literature. Data from focal follows of 113 mother–infant dyads in a remote area of Papua New Guinea, including parental and caretaker responses to infant ‘demands’ and patterns of breastfeeding, are presented and used to call into question the most common definition of ‘demand’ breastfeeding as a modality of infant feeding in which the infant is the locus of control.

The concept of ‘demand’ breastfeeding seems to have first appeared in the scientific literature in 1952 with Illingworth *et al*.’s paper on ‘self-demand feeding’ in a British maternity ward [2]. In that paper, ‘self-demand’ feeding is said to typify ‘primitive’ as opposed to ‘civilized’ communities, is said to lead to a higher incidence of successful lactation, and is contrasted with a ‘rigid feeding schedule’ [2:683]. In that same year, a paper by Hay *et al*. [3] that considered the potential difficulties for hospital wards of allowing women to breastfeed their babies *ad libitum* used the terms ‘self-demand’ and ‘on demand’ breastfeeding interchangeably. Although many more papers employing these concepts appeared subsequently, just over 30 years later, Lunn [4] published an important paper titled ‘Maternal nutrition and lactational infertility: the baby in the driving seat’. The central theme of the paper was that maternal nutritional status modulates the effectiveness of lactation as a contraceptive and posited that it does so by altering milk yields such that children of more poorly nourished mothers need to suckle ‘more frequently, longer and probably with more effort, in order to obtain the same amount of milk as those suckling well-fed mothers’ [4:47]. Although several studies have since corroborated the effect of maternal nutritional status and/or energy flux on the duration of lactational infecundity, mostly independent of suckling patterns [5–7], Lunn’s paper was nevertheless highly influential; not least, for situating the infant as the locus of control in the ‘on-demand’ breastfeeding process. As proposed by Lunn [4:46], ‘[i]f true “on-demand” feeding is being practiced by mothers, it is the baby who determines both the frequency and duration of breastfeeding’. Since the publication of Lunn’s paper in 1985, the concept of ‘demand’ breastfeeding, defined in this manner, has come to occupy a central place within the lactation literature, both scientific and popular.

Although what constitutes ‘demand breastfeeding’, beyond the central supposition that it is ‘infant-driven’ is ill-defined, the concept has been widely used in the literature of the demographic and biosocial sciences to characterize breastfeeding patterns most often seen in traditional, non-contracepting or ‘natural fertility’ populations [8–10]. The concept has been used to characterize feeding patterns of ‘traditional’ societies around the world, for example, in Africa [11–13], Australasia [6, 14, 15] and the Americas [16–18]. Adding to the haziness of the concept is that most of these sources state that infant feeding in these populations occurs ‘on demand’ but few to none actually report quantitative data on suckling frequency or duration. Consequently, it is unknown at present how much variability occurs among populations that are all said to practice ‘demand’ feeding.

The concept of ‘demand breastfeeding’ is commonly contrasted with the ‘scheduled’ feeding that often occurs in industrialized, ‘Western’ societies. ‘Scheduled’ feeding refers to a breastfeeding pattern that is controlled by the parent and is timed and restricted with respect to frequency and duration [19]. Popular parenting websites recommend that when fed on a schedule, newborns be fed once every 1.5–3 h, 2-month olds every 3–4 h and 6-month olds every 4–5 h [20]. In a study of 10 419 infants from the Avon Longitudinal Study of Parents and Children, Iacovou and Sevilla [21] found that mothers of children fed to a schedule reported higher measures of maternal well-being, including sufficient sleep, feeling confident and experiencing greater enjoyment than mothers who ‘demand’ fed. Schedule-fed children, however, experienced poorer cognitive function and academic outcomes. A large number of popular internet media sites concerned with parenting advocate for ‘demand’ feeding premised on the notion that ‘[e]volutionary, cross-cultural and clinical research suggests that babies were designed to feed on cue’ [22] and experience enhanced growth and cognitive development. Moreover, in the 21st century, pediatricians, even in industrialized nations, have begun to advocate for demand feeding, though these campaigns have met with only limited success due to competing economic and social demands on mothers [19]. Finally, a number of researchers have noted that deviations from originally pervasive patterns of infant rearing, that is, those that were predominant in the ‘environments of evolutionary adaptedness’ under which aspects of human biology evolved, can lead to pathological conditions, less than optimal developmental outcomes and elevated mortality risk [23–26]. These aspects of infant rearing include breastfeeding patterns but also co-sleeping and the like.

The notion that the baby, even in traditional societies, is ‘in the driver’s seat’, controlling the frequency and duration of breastfeeding bouts is likely to be incorrect for a number of both empirical and theoretical reasons. First, neonates simply do not possess the neuromuscular control to take the breast into the mouth at will. Therefore, although the baby may ‘demand’ the breast, it is the mother who ultimately decides whether and for how long feeding takes place. Second, according to evolutionary parent–offspring conflict theory, a subcategory of general parental investment theory, individual offspring are expected to seek maximal investment from parents, and it is against this backdrop of maximal investment-seeking by infants that parents make trade-off decisions about the opportunity costs and allocation of investment to individual children including the frequency and duration of breastfeeding [27, 28]. Trivers noted that the evolutionary basis for this conflict is that biological parents are related equally to all of their offspring and, thus, *ceteris paribus*, seek to invest in them equally [27]. Each individual offspring, however, is fully related to itself but only 50% on average to each of its siblings. Thus, the selection is expected to imbue each offspring with the desire to procure more parental resources for itself compared to its siblings [28]. Interestingly, Trivers used lactation as a specific example of parent–offspring conflict where, at some point in the process, natural selection begins to favor the mother halting parental investment in a given offspring even as natural selection acts on the offspring to favor it continuing to try to elicit as much additional parental investment as possible [27]. Therefore, although, as noted above, many popular media sites state that infants are ‘designed to feed on cue’, it is more likely that they are simply motivated to seek maximal investment.

This study uses focal follows on a sample of mother–infant dyads in a remote part of Papua New Guinea to quantify the types and frequency of responses of mothers and other caretakers to infant demands (i.e. bouts of fussing and crying).

## METHODOLOGY

### The study population

The study was conducted among the Au and Gnau people of Papua New Guinea. The Au and Gnau live in a remote area of Sandaun Province, West Sepik region of Papua New Guinea, 3°C south of the equator, in about 25 villages scattered throughout the southern foothills of the Torricelli Mountains. The area is primarily tropical rain forest. The Au and Gnau are forager–horticulturalists, subsisting primarily on starch harvested from semi-wild sago palms, although they obtain secondary staples such as taro and yams from small slash-and-burn gardens. Sago starch and tubers are generally consumed with a stew of leaves boiled in coconut cream. The stew sometimes also includes insect larvae and meat, typically from small nocturnally hunted marsupials, if available.

Previous research [29] has shown that the Au and Gnau have a total fertility rate of 6.1 and infant mortality averaging about 15%. Their relatively high infant mortality is likely related to their very low average birth weight of 2600 g and coupled with the presence of holoendemic malaria. The region occupied by the Au and Gnau is also widely known to be one of the most protein-restricted in Papua New Guinea. Infant and toddler growth from birth to age 5 years falls below the NCHS 5th percentile for both weight and height [29]. Au and Gnau also have the longest median duration of breastfeeding of any population studied to date, 43.7 months [6]. Bottles, pacifiers and infant formula were completely absent from the population at the time of this study. Thus, the quantification of ‘demand’ feeding and other infant pacification techniques is not confounded by the presence of these novel devices. Further ethnographic details about the Au and Gnau can be found in Tracer [30].

Criteria for inclusion in the present sample were that the infant was an overtly healthy singleton birth, aged between 1 week and 30 months, and currently still being breastfed.

### Data collection

Data for this study were collected during two field seasons in six Au villages (Anguganak, Bogasip, Brugap, Wulukum, Winaluk, and Musu) and two Gnau villages (Rauit and Maimbil). Focal follows were conducted on a sample of 113 mother–infant pairs by the principal investigator and three research assistants. Each focal follow involved accompanying the pair on their complete rounds, whether in the village or through the bush, for a period ranging from 4 to 6 h. In total, 470 h of focal follow observations, 54.5% during morning hours and 45.5% after noon, were recorded for the 113 pairs. Twelve times per hour (every 5 min), the type of task engaged in by the mother, the identities of all others accompanying her, and the proximity of the child to the mother (i.e. whether carried by mother, carried by another, on the ground, in line or sight of mother or not) were recorded. In addition, whenever ‘fussing’ was exhibited by the child, the response time, identity of responder and type of response was recorded. Each instance during which the child was given the breast either for feeding or pacification (a suckling ‘event’) was timed with a stopwatch and recorded to the nearest second.

Demographic and anthropometric variables were also collected for each mother and infant. Maternal variables included age (years), weight (kg), skinfold thickness at the triceps and subscapula (mm) and mid-arm and maximum calf circumferences (mm). Since most mothers do not know their ages, this variable could only be ascertained for 41 out of the 113 mothers. Infant variables collected included age (months), sex, birth order, weight (kg), recumbent length (mm), triceps skinfold thickness (mm) and mid-arm and head circumference (mm). Mothers’ weights were collected using a Seca standing scale, and infants’ weights were collected using a Seca pan scale. Infants’ recumbent lengths were collected using a baby board. All circumferences were measured using a flexible steel tape and skinfold thicknesses using Lange skinfold calipers.

## RESULTS

Table 1 gives descriptive statistics for the 113 mother–infant dyads. Mothers ranged from 19 to 41 years old with a mean age of 29. Their weight averaged 46.7 kg or roughly 102 lbs. As in an earlier and somewhat larger sample of Au women surveyed [6], this sample is relatively poorly nourished and tends to fall around the fifth percentile for anthropometric measures compared to published standards [31]. Infants ranged from 1 week to 2.5 years old with a mean of 11.7 months.

**Table 1.** Descriptive statistics for 113 mother–infant pairs^a^

Maternal variables				
Variable	Min	Max	Mean	SD
Age (years)^b^	19.0	41.0	29.1	6.8
Weight (kg)	34.8	59.8	46.7	5.7
Triceps skinfold (mm)	2.0	12.3	6.3	2.0
Subscapular skinfold (mm)	4.0	18.0	10.3	2.9
Mid-arm circumference (mm)	19.1	27.1	22.6	1.8
Max calf circumference (mm)	26.1	37.4	31.1	2.2

Infant variables				
Age (months)	0.20	30.0	11.7	6.4
Birth order (#)	1.0	9.0	3.3	1.9
Weight (kg)	2.3	11.1	6.9	1.6
Recumbent length (mm)	454.0	835.0	665.3	71.4
Triceps skinfold (mm)	3.0	10.0	6.1	1.5
Mid-arm circumference (mm)	9.2	15.3	12.6	1.2
Head circumference (mm)	25.1	59.5	42.9	3.6

^a^Due to some missing values actual *n*’s range from 108 to 113 except for maternal age.

^b^Since most mother’s do not know their age, this variable had the most missing values; *n* = 41.

In total, 470 h of focal follows were conducted among the 113 mother–infant dyads. Over the 470 h, 415 suckling events were recorded. Table 2 shows the characteristics of all breastfeeding bouts recorded during these follows for all mother–infant pairs, including the number of events per hour and the timed duration in seconds of each event. On average, infants were breastfed 3.6 times per hour and the duration of each event was approximately 2.3 min per feed.

**Table 2.** Breastfeeding characteristics among 113 mother–infant pairs

Variable	Minimum	Maximum	Mean	SD
BF events (#/h)	0.00	18.0	3.6	4.2
BF duration (s)	30.63	401.2	137.5	78.7

**Figure 1.**
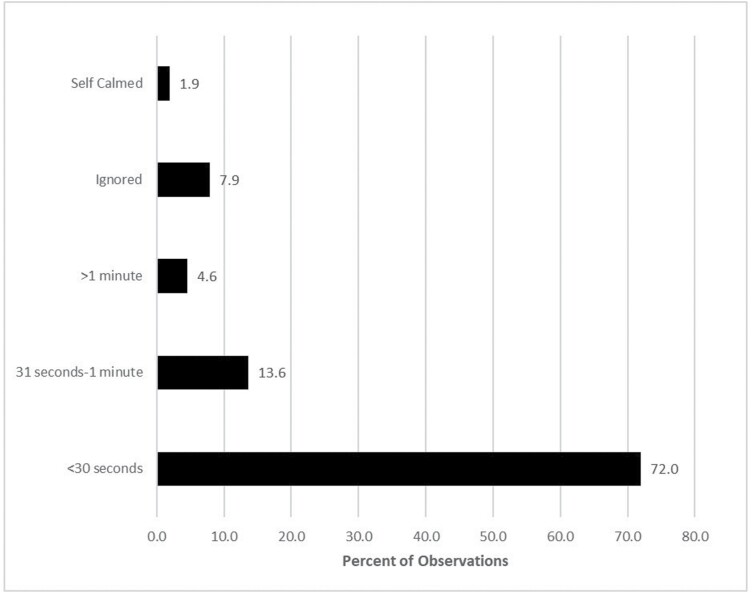
Response time of caretakers to fussing or crying infants.

Figure 1 shows the amount of time that it took for caretakers to respond to fussing and crying infants. In only 9.8% of cases, fussing or crying infants were not attended to and either ignored or allowed to self-calm. In the other 90.2% of cases, the vast majority of infants were attended to in less than 1 min. This demonstrates that the Au and Gnau display very high levels of parental solicitude and responsiveness toward their infants.

**Figure 2.**
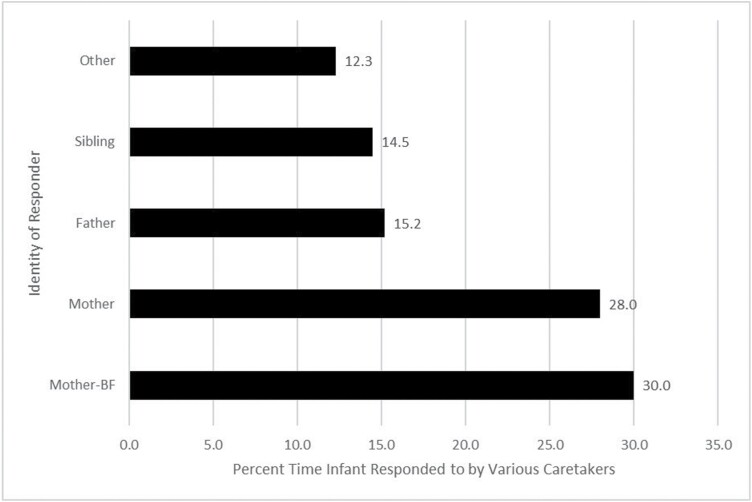
Percent time various caretakers respond to fussing or crying infants. If the mother was the responder, percent time responded by offering the breast versus other modalities of calming.

Figure 2 shows the percent time that various caretakers responded to fussing and crying offspring and, if the mother responded, the percent time that she responded by offering the breast. The figure shows that the mother responded to fussing and crying children 58% of the time. Other responders included the father 15.2% of the time, a sibling 14.5% of the time, and others such as cousins or other village mates 12.3% of the time. Interestingly, of the 58% of the time that mothers responded to their fussing or crying children, they offered them the breast just 52% of the time. The other 48% of maternal responses mostly involved consoling the child by patting or talking to them, sometimes reassuringly and, occasionally, scoldingly.

Table 3 shows Pearson correlation coefficients between various maternal and child demographic and z-scored anthropometric variables with suckling frequency and duration. This analysis was conducted in an attempt to identify possible determinants of suckling frequency and duration in the sample. As seen in the table, neither maternal age nor any anthropometric characteristics were significantly associated with the frequency or duration of suckling. Infant’s age was, however, significantly and negatively associated with suckling event duration as were three out of five of the z-scored anthropometric dimensions measured. The shorter event duration among older children in the sample is likely a product of the introduction of some supplementary foods to these older children. The negative association of z-scored weight, recumbent length and mid-upper arm circumference with suckling duration may indicate that mothers attempt to increase investment and, thus, the probability of survival, among children in relatively poorer condition by breastfeeding them for longer periods.

**Table 3.** Pearson correlation coefficients of maternal and child demographic and z-scored anthropometric variables with sucking frequency and duration

Maternal variables		
	Event frequency	Event duration
Age (years)	0.182	0.121
Weight	−0.059	0.022
Triceps skinfold	−0.063	−0.145
Subscapular skinfold	−0.029	−0.134
Mid-upper arm circumference	−0.004	−0.030
Max calf circumference	−0.120	0.088

Infants variables		
	Event frequency	Event duration
Age (months)	0.003	−0.337^**^
Sex	−0.089	0.017
Weight	−0.067	−0.357^**^
Recumbent length	0.012	−0.310
Triceps skinfold	−0.001	−0.114
Mid-upper arm circumference	−0.119	−0.242^*^
Head circumference	−0.009	−0.143

^*^ *P* < 0.05;

^**^ *P* < 0.01.

In an effort to further examine whether the child or mother plays the dominant role as the driver of breastfeeding behavior, a logistic regression of maternal responses to infant’s ‘demands’ (i.e. fussing and crying) with age, sex and BMI of the child as well as time in minutes since the child was previously breastfed was conducted. The maternal response was coded dichotomously as consoled the child by some means other than breastfeeding (coded ‘0’) or responded by breastfeeding (coded ‘1’). If the child was the predominant driver of breastfeeding behavior, it might be expected that mothers would tend to breastfeed their offspring whenever they fussed or cried independently of the amount of time that had elapsed since last breastfeeding. If, however, the mother was the predominant driver of breastfeeding initiation, then it is expected that mothers would tend to respond to their offspring with behaviors other than breastfeeding the more recently the child had last been breastfed. The results of this analysis are shown in Table 4. The overall regression model is highly significant at the *P* < 0.001 level. Of the three variables in the analysis, time since last breastfeeding has the most significant relationship to the dichotomous response variable. That is, the more recently the child was last offered the breast, the more likely it is that the mother responded to the current bout of fussing or crying by attempting to console the child by some means *other* than breastfeeding. In addition, both age and nutritional status of the child (as indicated by BMI) are each significantly and negatively related to the response variable; that is, the younger or less well-nourished the child, the more likely the mother is to respond to the child’s fussing and crying by breastfeeding.

**Table 4.** Logistic regression of maternal responses to infant’s ‘demands’ (i.e. fussing and crying) with age, sex and BMI of the child and time (minutes) since the child was last breastfed (TSLBF)

Variable	Coefficient	SE
Age (months)	−0.047	0.020^*^
Sex (m/f)	0.045	0.223
BMI	−0.113	0.056^*^
TSLBF (min)	0.025	0.004^***^

Dichtomous maternal responses were coded as responded to child by means other than breastfeeding (‘0’), responded by breastfeeding (‘1’).

^*^ *P* < 0.05;

^**^ *P* < 0.01;

^***^ *P* < 0.001.

**Figure 3.**
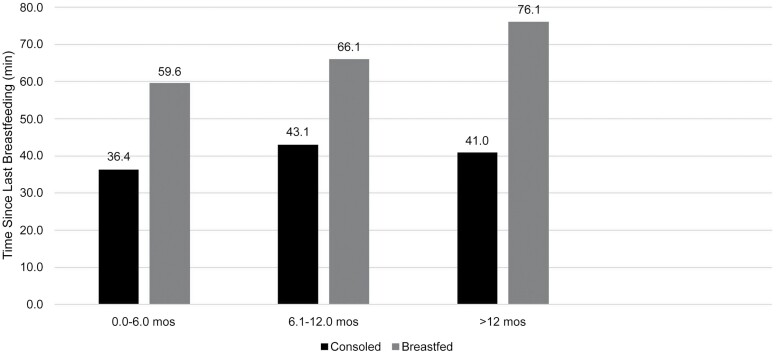
Type of response (‘consoled’ by a means other than breastfeeding vs infant is ‘breastfed’) by child’s age category and time since last breastfeeding.

Figure 3 shows the type of response to infant ‘demands’ (fussing and crying) by the child’s age category and time since last breastfeeding. As in the prior logistic regression, the type of response is dichotomized into ‘consoled’ by means other than breastfeeding and ‘breastfed’. The figure illustrates that, in every age category, mothers are more likely to breastfeed their fussing and crying infants the longer it has been since the last breastfeeding. In the youngest age category, birth–6 months, mothers tend to respond to infant demands with breastfeeding when it had been approximately 1 h on average since the child was last breastfed. In the older than 6-month to 1-year age category, this interval rises by 6 min, and in the oldest age category (>12 months), by another 10 min.

## DISCUSSION AND CONCLUSIONS

The concept of ‘demand’ breastfeeding first appeared in the scientific literature in 1952 with Illingworth *et al*.’s paper on ‘self-demand feeding’ in a British maternity ward [2]. In that paper, ‘self-demand’ feeding was said to typify ‘primitive’ as opposed to ‘civilized’ communities, was shown to lead to a higher incidence of successful lactation, and was contrasted with a ‘rigid feeding schedule’ [2:683]. Just over 30 years later, Lunn’s paper titled ‘Maternal nutrition and lactational infertility: the baby in the driving seat’ similarly situated the infant in traditional societies as the locus of control in the ‘demand’ breastfeeding process [4]. Based on the knowledge that very young infants are incapable of taking the breast into the mouth at will and the logic of evolutionary parental investment theory, which posits that parents make investment decisions in individual offspring, even as those infants seek maximal investment, the present study sought to test the notion that infants are the locus of control in the ‘demand’ feeding process.

The results of the present study demonstrate that the Au/Gnau pattern of breastfeeding is characterized by short and frequent feeds. This finding is in stark contrast to common patterns of scheduled feeding in industrialized ‘Western’ societies, which seldom exceed a single feed at most every 2 h. Therefore, it is unquestionable that feeding in this ‘traditional’ society is more frequent than typically occurs in societies practicing ‘scheduled’ feeding. What is questionable, however, is whether this pattern is truly infant-driven, as proposed by Illingworth, Lunn, and others. Rather, in response to infant demands, indexed by bouts of fussing and/or crying, Au and Gnau mothers offer the breast just 52% of the time. Other responses ranging from ignoring, patting or speaking to the child are employed the other 48% of the time. Furthermore, analyses showing that Au and Gnau mothers tend to respond to their offsprings’ demands by means other than breastfeeding the more recently the child has been breastfed, suggests that it is the mother rather than the infant that is the locus of control in the breastfeeding process.

Overall, this study suggests that rather than being in the ‘driver’s seat’, the infant is actually more like the proverbial ‘back seat’ driver—making near-constant suggestions to the parent who then responds to those suggestions, inwhatever way is deemed appropriate at the time given perceived opportunity costs, benefits and tangible constraints. This study refutes the notion that the infant is the locus of control in the ‘demand’ feeding process. Rather, it suggests that, as predicted by evolutionary parental investment theory, parents make trade-off decisions about the allocation of investment to individual children even as those offspring seek maximal parental investment. Given that this is the case, the study suggests that the form of infant feeding practiced in traditional societies is better referred to as ‘response’ feeding, placing the parent as the locus of decision-making, rather than ‘demand’ feeding.
